# Supplementary material for: Modeling glioblastoma heterogeneity as a dynamic network of cell states
Source: Mol Syst Biol. 2021 Sep 16;17(9):e10105. doi: 10.15252/msb.202010105 (PMC8444284; doi:10.15252/msb.202010105)
Supplement: Supplementary file 6 — Source Data for Figure 5 [file MSB-17-e10105-s004.zip › Figure5A_sourcedata/GSEA_3071/hallmarks_stateII.GseaPreranked.1623742536692/HALLMARK_MYC_TARGETS_V1.html]

Details for gene set HALLMARK\_MYC\_TARGETS\_V1[GSEA]

|  || Dataset | state23071 |
| Phenotype | NoPhenotypeAvailable |
| Upregulated in class | na\_pos |
| GeneSet | HALLMARK\_MYC\_TARGETS\_V1 |
| Enrichment Score (ES) | 0.48698708 |
| Normalized Enrichment Score (NES) | 3.0696926 |
| Nominal p-value | 0.0 |
| FDR q-value | 0.0 |
| FWER p-Value | 0.0 |
Table: GSEA Results Summary

  

Fig 1: Enrichment plot: HALLMARK\_MYC\_TARGETS\_V1      
 Profile of the Running ES Score & Positions of GeneSet Members on the Rank Ordered List

  

| PROBE | GENE SYMBOL | GENE\_TITLE | RANK IN GENE LIST | RANK METRIC SCORE | RUNNING ES | CORE ENRICHMENT || 1 | TYMS |  |  | 8 | 1.665 | 0.0262 | Yes |
| 2 | USP1 |  |  | 31 | 1.357 | 0.0437 | Yes |
| 3 | RRM1 |  |  | 37 | 1.310 | 0.0647 | Yes |
| 4 | MCM7 |  |  | 45 | 1.273 | 0.0845 | Yes |
| 5 | CCNA2 |  |  | 53 | 1.230 | 0.1037 | Yes |
| 6 | PCNA |  |  | 58 | 1.214 | 0.1232 | Yes |
| 7 | CDC45 |  |  | 59 | 1.212 | 0.1438 | Yes |
| 8 | MAD2L1 |  |  | 61 | 1.199 | 0.1639 | Yes |
| 9 | MCM5 |  |  | 74 | 1.128 | 0.1800 | Yes |
| 10 | MCM4 |  |  | 81 | 1.110 | 0.1973 | Yes |
| 11 | DUT |  |  | 118 | 1.009 | 0.2054 | Yes |
| 12 | CDK4 |  |  | 137 | 0.986 | 0.2176 | Yes |
| 13 | RANBP1 |  |  | 149 | 0.958 | 0.2311 | Yes |
| 14 | CTPS1 |  |  | 162 | 0.934 | 0.2440 | Yes |
| 15 | MCM6 |  |  | 176 | 0.906 | 0.2561 | Yes |
| 16 | KPNB1 |  |  | 186 | 0.891 | 0.2689 | Yes |
| 17 | TFDP1 |  |  | 209 | 0.866 | 0.2781 | Yes |
| 18 | NOP56 |  |  | 250 | 0.824 | 0.2821 | Yes |
| 19 | EIF2S1 |  |  | 282 | 0.776 | 0.2875 | Yes |
| 20 | POLE3 |  |  | 297 | 0.758 | 0.2968 | Yes |
| 21 | DEK |  |  | 302 | 0.754 | 0.3086 | Yes |
| 22 | HPRT1 |  |  | 351 | 0.709 | 0.3086 | Yes |
| 23 | RFC4 |  |  | 385 | 0.686 | 0.3120 | Yes |
| 24 | PA2G4 |  |  | 412 | 0.672 | 0.3168 | Yes |
| 25 | KPNA2 |  |  | 420 | 0.669 | 0.3264 | Yes |
| 26 | RAN |  |  | 424 | 0.667 | 0.3370 | Yes |
| 27 | ODC1 |  |  | 430 | 0.664 | 0.3470 | Yes |
| 28 | BUB3 |  |  | 441 | 0.657 | 0.3556 | Yes |
| 29 | SNRPD1 |  |  | 523 | 0.615 | 0.3458 | Yes |
| 30 | CDK2 |  |  | 530 | 0.612 | 0.3546 | Yes |
| 31 | SRM |  |  | 535 | 0.610 | 0.3640 | Yes |
| 32 | PPM1G |  |  | 539 | 0.609 | 0.3736 | Yes |
| 33 | NPM1 |  |  | 544 | 0.605 | 0.3828 | Yes |
| 34 | NOLC1 |  |  | 546 | 0.603 | 0.3928 | Yes |
| 35 | SERBP1 |  |  | 562 | 0.593 | 0.3991 | Yes |
| 36 | HSPD1 |  |  | 606 | 0.576 | 0.3981 | Yes |
| 37 | DDX21 |  |  | 607 | 0.575 | 0.4078 | Yes |
| 38 | SRPK1 |  |  | 610 | 0.574 | 0.4171 | Yes |
| 39 | FBL |  |  | 612 | 0.573 | 0.4265 | Yes |
| 40 | CAD |  |  | 620 | 0.569 | 0.4344 | Yes |
| 41 | CCT5 |  |  | 662 | 0.548 | 0.4335 | Yes |
| 42 | PRPS2 |  |  | 667 | 0.546 | 0.4417 | Yes |
| 43 | PABPC4 |  |  | 677 | 0.538 | 0.4486 | Yes |
| 44 | ABCE1 |  |  | 716 | 0.527 | 0.4480 | Yes |
| 45 | MCM2 |  |  | 734 | 0.521 | 0.4526 | Yes |
| 46 | PTGES3 |  |  | 763 | 0.512 | 0.4543 | Yes |
| 47 | EIF3B |  |  | 774 | 0.506 | 0.4603 | Yes |
| 48 | MYC |  |  | 790 | 0.502 | 0.4651 | Yes |
| 49 | CDC20 |  |  | 844 | 0.486 | 0.4601 | Yes |
| 50 | SRSF2 |  |  | 941 | 0.461 | 0.4438 | Yes |
| 51 | SNRPA1 |  |  | 954 | 0.458 | 0.4486 | Yes |
| 52 | CCT4 |  |  | 999 | 0.445 | 0.4451 | Yes |
| 53 | TRIM28 |  |  | 1004 | 0.444 | 0.4516 | Yes |
| 54 | SRSF7 |  |  | 1071 | 0.424 | 0.4423 | Yes |
| 55 | PABPC1 |  |  | 1075 | 0.422 | 0.4487 | Yes |
| 56 | CCT2 |  |  | 1108 | 0.415 | 0.4477 | Yes |
| 57 | TCP1 |  |  | 1116 | 0.414 | 0.4530 | Yes |
| 58 | UBA2 |  |  | 1131 | 0.412 | 0.4565 | Yes |
| 59 | EIF3J |  |  | 1159 | 0.405 | 0.4566 | Yes |
| 60 | HNRNPD |  |  | 1177 | 0.401 | 0.4591 | Yes |
| 61 | LSM2 |  |  | 1178 | 0.401 | 0.4659 | Yes |
| 62 | EIF1AX |  |  | 1198 | 0.397 | 0.4679 | Yes |
| 63 | SNRPA |  |  | 1213 | 0.394 | 0.4711 | Yes |
| 64 | IMPDH2 |  |  | 1228 | 0.391 | 0.4742 | Yes |
| 65 | POLD2 |  |  | 1237 | 0.389 | 0.4788 | Yes |
| 66 | SF3A1 |  |  | 1245 | 0.387 | 0.4836 | Yes |
| 67 | SNRPD3 |  |  | 1303 | 0.377 | 0.4758 | Yes |
| 68 | NME1 |  |  | 1343 | 0.368 | 0.4722 | Yes |
| 69 | RSL1D1 |  |  | 1373 | 0.361 | 0.4711 | Yes |
| 70 | LDHA |  |  | 1374 | 0.361 | 0.4772 | Yes |
| 71 | ACP1 |  |  | 1396 | 0.358 | 0.4780 | Yes |
| 72 | EIF3D |  |  | 1405 | 0.357 | 0.4821 | Yes |
| 73 | C1QBP |  |  | 1433 | 0.351 | 0.4813 | Yes |
| 74 | CCT3 |  |  | 1446 | 0.347 | 0.4841 | Yes |
| 75 | PRDX4 |  |  | 1470 | 0.344 | 0.4842 | Yes |
| 76 | CSTF2 |  |  | 1498 | 0.341 | 0.4832 | Yes |
| 77 | ETF1 |  |  | 1507 | 0.339 | 0.4870 | Yes |
| 78 | FAM120A |  |  | 1642 | 0.318 | 0.4588 | No |
| 79 | SF3B3 |  |  | 1651 | 0.316 | 0.4622 | No |
| 80 | G3BP1 |  |  | 1661 | 0.315 | 0.4653 | No |
| 81 | EIF4H |  |  | 1767 | 0.303 | 0.4441 | No |
| 82 | EIF4A1 |  |  | 1773 | 0.302 | 0.4480 | No |
| 83 | RAD23B |  |  | 1777 | 0.301 | 0.4523 | No |
| 84 | RUVBL2 |  |  | 1779 | 0.301 | 0.4572 | No |
| 85 | NOP16 |  |  | 1809 | 0.298 | 0.4550 | No |
| 86 | ILF2 |  |  | 1833 | 0.294 | 0.4542 | No |
| 87 | SRSF3 |  |  | 1834 | 0.294 | 0.4592 | No |
| 88 | RNPS1 |  |  | 1841 | 0.293 | 0.4627 | No |
| 89 | GLO1 |  |  | 1867 | 0.290 | 0.4613 | No |
| 90 | CYC1 |  |  | 1938 | 0.281 | 0.4485 | No |
| 91 | PSMB2 |  |  | 1950 | 0.279 | 0.4505 | No |
| 92 | DHX15 |  |  | 1985 | 0.275 | 0.4467 | No |
| 93 | PSMD1 |  |  | 1993 | 0.273 | 0.4495 | No |
| 94 | NCBP1 |  |  | 1999 | 0.273 | 0.4529 | No |
| 95 | AP3S1 |  |  | 2014 | 0.271 | 0.4540 | No |
| 96 | EEF1B2 |  |  | 2041 | 0.268 | 0.4520 | No |
| 97 | RRP9 |  |  | 2055 | 0.267 | 0.4533 | No |
| 98 | PPIA |  |  | 2072 | 0.265 | 0.4538 | No |
| 99 | PGK1 |  |  | 2126 | 0.260 | 0.4449 | No |
| 100 | XRCC6 |  |  | 2180 | 0.254 | 0.4359 | No |
| 101 | PCBP1 |  |  | 2192 | 0.253 | 0.4375 | No |
| 102 | CANX |  |  | 2427 | -0.274 | 0.3835 | No |
| 103 | NDUFAB1 |  |  | 2716 | -0.320 | 0.3168 | No |
| 104 | SMARCC1 |  |  | 2837 | -0.342 | 0.2925 | No |
| 105 | UBE2E1 |  |  | 2904 | -0.356 | 0.2820 | No |
| 106 | LSM7 |  |  | 3028 | -0.379 | 0.2576 | No |
| 107 | NCBP2 |  |  | 3588 | -0.598 | 0.1278 | No |
Table: GSEA details [plain text format]

  

Fig 2: HALLMARK\_MYC\_TARGETS\_V1: Random ES distribution      
 Gene set null distribution of ES for **HALLMARK\_MYC\_TARGETS\_V1**
